# Completion Rates of Advance Directives in a Trauma Emergency Room: Association with Age

**DOI:** 10.1155/2021/5537599

**Published:** 2021-04-20

**Authors:** Jan A. Graw, René Burchard

**Affiliations:** ^1^Department of Anesthesiology and Operative Intensive Care Medicine CCM/CVK, Charité–Universitätsmedizin Berlin, Corporate Member of Freie Universität Berlin, Humboldt-Universität zu Berlin, Berlin Institute of Health, Berlin, Germany; ^2^Berlin Institute of Health (BIH), Berlin, Germany; ^3^Department of Trauma and Orthopaedic Surgery, Lahn-Dill-Kliniken, Dillenburg, Germany; ^4^Department of Orthopedics and Trauma Surgery, University of Giessen and Marburg, Marburg, Germany; ^5^School of Science and Technology, University of Siegen, Siegen, Germany

## Abstract

*Introduction* An advance directive (AD) is a written legal document in which a person can express wishes and preferences for medical treatment for the moment when that person is no longer able to make medical decisions because of a serious illness or injury. While ADs have emerged in public, it is unclear, how many adults in Germany have completed an AD, and frequencies differ among different patient cohorts and medical settings. The aim of this study was to evaluate how many patients visiting a trauma emergency room (ER) in an academic teaching hospital had completed an AD. Furthermore, patient characteristics were compared between patients who had completed an AD and those who had not completed an AD. *Methods*. Patients with a traumatic injury or disease who attended the ER of an academic teaching hospital in the period from October 2015 to March 2016 (*n* = 499) were surveyed for completion rates of ADs. *Results*. Prior to their visit to the ER, 12.8% of the included patients possessed a completed AD. Patients with a completed AD had a higher age (median age: 54 (IQR: 34–66) vs. 35 (IQR: 25–50) *p* < 0.001) and were less often living in an urban residential location (UR) (UR: 23.5% vs. 39.4%, *p*=0.029). Groups did not differ between sex (*p*=0.115), frequencies of high school graduates (*p*=0.482), and possession of a private health insurance (*p*=0.072), disability insurance (*p*=0.291), or an accident insurance (*p*=0.790). *Conclusion*. Completion rates of ADs remain low among patients visiting an ER of an academic teaching hospital in Germany. Increasing age but not factors such as sex, educational background, or insurance status were associated with a higher frequency of completed ADs.

## 1. Introduction

The prevention, cure, and care of ill or injured patients using diagnostic and therapeutic medical measures are based on two fundamental principles of medicine: the medical indication and the patient's consent. Involvement and participation of patients in medical decision-making is known as “shared decision-making” or “participatory decision-making” [1]. For autonomous and self-determined decision-making regarding any diagnostic or therapeutic medical activities, the patient must have the capacity to act and decide momentously [2, 3]. In particular, in situations of severe and critical illness, this capacity is often lost, and the patient cannot actively oversee and participate in the medical decision-making process [4–6].

An advance directive is a written legal document in which a person can express wishes and preferences for medical treatment for the moment when that person is no longer able to make medical decisions because of a serious illness or injury. Therefore, an advance directive can preserve a person's autonomy and self-determination once decision-making capacity is lost. In the last two decades of this century, many countries have approved principles of advance directives and anticipated shared decision-making situations at the end of life by their legislative authorities [7, 8]. In Germany, the first law to legally regulate advance directives was enacted in 2009 [8]. Subsequently, a physician and also a patient's attorney or surrogate decision-maker were not anymore allowed to overrule a patient's pre-emptively formulated will and preference for treatment decisions when this patient had lost his or her decision-making capacity [8].

While advance directives have more and more emerged in public, it is unclear, how many adults in Germany have completed such a document. There are only few data reporting incidences of advance directives in the public, and frequencies differ among different patient cohorts and medical settings [5, 9, 10].

For this study, we reanalyzed data of a previously reported survey with respect to a nonreported survey question whether the interviewed patient had previously completed an advance directive [11]. The aim of this study was to evaluate how many patients visiting a trauma emergency room in an academic teaching hospital had completed an advance directive. Furthermore, characteristics of patients who had completed an advance directive were compared to characteristics of patients who had not completed an advance directive.

## 2. Methods

### 2.1. Study Design and Setting

A survey to study the indicated trauma emergency department utilization was offered to all patients with a traumatic injury or disease who attended the emergency room of the department of trauma surgery at an academic teaching hospital in the period from October 2015 to March 2016 [11]. The patient cohort that did not have a formal referral of a community-based physician or general practitioner (GP) was surveyed. In addition to the already reported data, patients were asked whether they had completed an advance directive. Surveys with multiple participations, surveys with a missing response whether an advance directive was completed, and surveys of patients of an age <18 years or of patients admitted by ambulance or with a Manchester Triage Score below four were excluded.

Written informed consent was obtained from all study participants before participation. All questionnaires were collected anonymously through a voting box system. The study was approved by the Medical Ethics Committee of the Medical Council Westphalia-Lippe (number: 2015-497-f-S).

### 2.2. Outcome

Description of characteristics of adult patients who attend an emergency room of an academic teaching hospital for a traumatic injury or disease and who had completed an advance directive compared with patients who had not completed an advance directive.

### 2.3. Statistics

Results are expressed in absolute numbers and frequencies (%) or mean and standard deviation (SD) unless indicated otherwise. Data were analyzed with statistical software package SPSS® version 26 (IBM, Armonk, North Castle, New York, USA). Continuous variables of two independent groups were compared using nonparametric Wilcoxon–Mann–Whitney test. Categorical data were compared using Chi-square test with *z*-test to compare cell counts across the columns. All tests were conducted in the area of exploratory data analysis. Therefore, no adjustments for multiple testing have been made. A *p* value of less than 0.05 was considered significant.

## 3. Results

**Figure 1:**
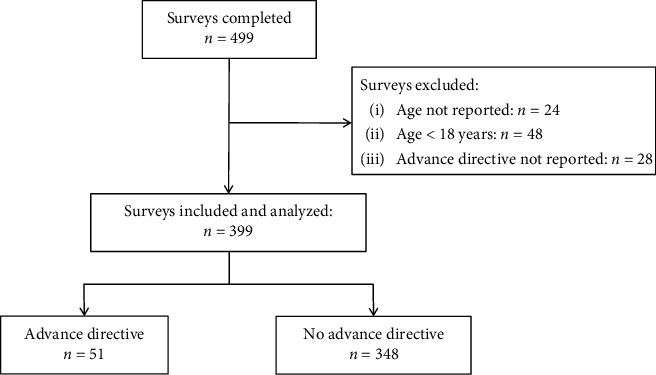
Consort diagram.

As previously reported, in the period from October 2015 to March 2016, 499 patients visiting the emergency room of the department of trauma surgery without a formal referral of a community-based physician or general practitioner (GP) completed the survey [11]. These were 32.9% of all eligible patients [11]. Fifty-two surveys of patients who did not respond to the question of their age or whether they had completed an advance directive (10.4%) and 48 surveys of patients <18 years old (9.6%) were excluded. Therefore, 399 surveys were included in the analysis (Figure 1).

### 3.1. Respondent Basic Characteristics

**Figure 2:**
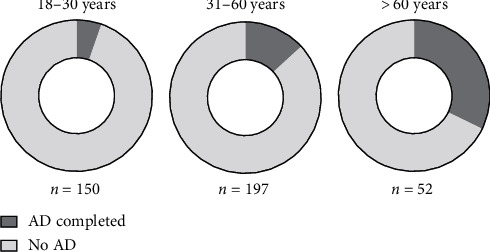
Percentage of patients with a completed advance directive separated into three different age groups: 18–30 years, 31–60 years, and older than 60 years.

Fifty-one (12.8%) patients possessed a completed advance directive prior to their visit to the emergency room. Characteristics of patients with a completed advance directive and those without an advance directive are presented in Table 1. The patients had a median age of 37 years (IQR: 25–52), and 62.9% (*n* = 251) of all patients were male. There was no statistical difference in the patients' sex between the compared groups (Table 1). Patients with an advance directive were older compared to patients without an advance directive (median age with an advance directive: 54 (IQR: 34–63) vs. median age without an advance directive: 35 (IQR: 25–50, *p* < 0,001). Patients above the age of 60 years had more often completed an advance directive compared to patients with an age of and below 60 years (advance directive completed and >60 years: 32.7% (*n* = 17) vs. advance directive completed and ≤60 years: 9.8% (*n* = 34), *p* < 0,001).  Figure 2 presents the percentages of patients who had completed an advance directive separated into age groups from 18 to 30, 31–60, and >60 years.

### 3.2. Respondent Characteristics with Regard to Insurance Status and Living Location

There were no differences in the frequencies of possession of a private health insurance, disability insurance, or an accident insurance among patients who had completed an advance directive and patients who had not completed an advance directive (Table 1). Among patients who had completed an advance directive, 23.5% (*n* = 12) were living in an urban residential location compared to 39.4% (*n* = 137) of patients who had not completed an advance directive (*p*=0.029, Table 1).

### 3.3. Respondent Characteristics with Regard to Educational Background and Regular Physical Activity

Regarding the educational background, there was no statistical significant difference between the relative frequencies of high school graduates between patients who had completed an advance directive and those who had not completed an advance directive (Table 2). Furthermore, there was no statistical significant difference between patients who had completed an advance directive and those who had not completed an advance directive with regard to regular physical activity (Table 2).

## 4. Discussion

This cross-sectional study revealed that only 12.8% of patients visiting the emergency room of an academic teaching hospital in Germany with a traumatic injury or disease had previously completed an advance directive. It appeared that increasing age but not factors such as gender, educational background or insurance status, and living conditions were associated with a higher frequency of completed advance directives.

Although it was assumed that the number of inhabitants in Germany that complete some form of advance directive would increase after a law on advance directives was enacted in 2009, completion rates of advance directives appear to remain generally low at a first sight [5, 12, 13]. However, here we found that with increasing age, completion rates of advance directives increased in patients with an acute traumatic injury or disease. Apart from factors such as familiarity with the instrument of an advance directive or legal consultations or advices during legal testament preparations, age seems to robustly impact completion rates of advance directives in many different patient populations [12, 14–16]. Patients with a traumatic injury and an according injury severity that allows self-admittance to a trauma emergency room are frequently younger compared to a general patient population that is admitted to an emergency room with medical problems. However, our results are in line with previously found data [17].

A second factor known to be generally associated with the completion of an advance directive is the presence of a life-threatening disease or personal life-and-death experiences [18–21]. However, even in cancer patients, completion rates of advance directives are lower in comparison with the general population for certain patient cohorts [22, 23]. In this study, patients with traumatic injuries that allowed self-admittance to the trauma emergency room were surveyed, and patients admitted by ambulance or with a high Manchester triage score were excluded. Therefore, the incidence of acutely life-threatening diseases and injuries in this patient cohort might be significantly lower compared to incidences in surveys performed in a general emergency room, in outpatient clinics, or in community-based private practices [12].

Data on an association between completion rates of advance directives and gender or the level of education are reported from the United States and some European countries [24–27]. The gender effect was also seen in a recent cross-sectional study among more than 2000 people in Switzerland [28]. The authors speculated that reasons for a higher completion rate of advance directives among women might be associated with their higher life expectation and therefore a higher chance to survive their spouse [28]. Experiencing death in close relatives or caring for a dying person is a known trigger for people to start thinking about end-of-life issues and preparing an advance directive [21]. The association of advance directive completions rates with a higher level of education might partly be explained by the readability of state-sponsored advance directive forms. A structured analysis in the United States revealed that the readability of state-sponsored advance directive forms in most cases exceeded the average reading skill level of most adults [29]. In the patient cohort of this study, we could detect neither a gender effect nor a difference in the level of education between patients with a completed advance directive and patients without an advance directive. Besides, the low median age of the patients in our studied cohort, the low overall completion rate of advance directives, and a generally high educational level with more than 90% of patients having graduated from secondary schools or possessing A-levels might explain our findings. Furthermore, the current survey did not include data on marriage status. Adjustments for marriage status should be made in further studies addressing gender effects associated with completion rates of advance directives.

The trauma department that oversees the trauma emergency room where the survey was conducted has a special focus on sports traumatology. Therefore, this study is limited by a potential selection bias including a low median age of the surveyed patients in addition to the single-center design. In addition, no data were obtained on comorbidities or the general health status of the individual patients. Furthermore, this study is a subgroup analysis of a survey not intended to primarily investigate factors associated with advance directives. Besides the high number of patients not fully completing the initial survey, one-fifth of the surveys for this analysis had to be excluded because of missing data on advance directives, age, or because patients were <18 years old.

## 5. Conclusion

Completion rates of advance directives remain low among patients visiting an emergency room of an academic teaching hospital in Germany with a traumatic injury or disease. Increasing age but not factors such as sex, educational background or insurance status, and living conditions were associated with a higher frequency of completed advance directives in this specific patient population.

## Figures and Tables

**Table 1:** Patient characteristics.

	All (*n* = 399)		Advance directive completed (*n* = 51)		No advance directive (*n* = 348)		*p* value
Age, years (±SD)	40.5	(±17.6)	51.8	(±18.9)	38.8	(±16.7)	<0.001
Male sex, *n* (%)	251	(62.9)	27	(52.9)	224	(64.4)	0.115
Insurance type, *n* (%)							
Private health	30	(7.5)	7	(13.7)	23	(6.6)	0.072
Disability	126	(31.6)	13	(25.5)	113	(32.5)	0.291
Accident	212	(53.1)	27	(52.9)	185	(53.2)	0.790

Urban residential location, *n* (%)	149	(37.3)	12	(23.5)	137	(39.4)	0.029

Data are expressed as mean (±standard deviation (SD)) or frequencies (%), as appropriate.

**Table 2:** Level of education and weekly physical activity.

	Advance directive completed (*n* = 51)		No advance directive (*n* = 348)		*p* value
Level of education, *n* (%)					
No graduation or not responded	4	(7.8)	21	(6.0)	
Secondary school	35	(68.6)	217	(62.4)	0.482
High school graduation	12	(23.5)	110	(31.6)	

Weekly physical activity, *n* (%)	27	(52.9)	236	(67.8)	0.044

## Data Availability

The datasets used and/or analyzed during the current study are available from the corresponding author on reasonable request.

## References

[1] Charles C., Gafni A., Whelan T. (1997). Shared decision-making in the medical encounter: what does it mean? (or it takes at least two to tango). *Social Science & Medicine*.

[2] Palazzani L. (2004). Advance directives and living wills. *NeuroRehabilitation*.

[3] Di Paolo M., Gori F., Papi L., Turillazzi E. (2019). A review and analysis of new Italian law 219/2017: ‘provisions for informed consent and advance directives treatment’. *BMC Medical Ethics*.

[4] Cohen S., Sprung C., Sjokvist P. (2005). Communication of end-of-life decisions in European intensive care units. *Intensive Care Medicine*.

[5] Graw J. A., Spies C. D., Wernecke K. D., Braun J. P. (2012). Managing end-of-life decision making in intensive care medicine-a perspective from Charite Hospital, Germany. *PLoS One*.

[6] Sprung C. L., Ricou B., Hartog C. S. (2019). Changes in end-of-life practices in European intensive care units from 1999 to 2016. *Jama*.

[7] Sulmasy D. P. (2018). Italy’s new advance directive law. *JAMA Internal Medicine*.

[8] Wiesing U., Jox R. J., Hessler H.-J., Borasio G. D. (2010). A new law on advance directives in Germany. *Journal of Medical Ethics*.

[9] Halpern N. A., Pastores S. M., Chou J. F., Chawla S., Thaler H. T. (2011). Advance directives in an oncologic intensive care unit: a contemporary analysis of their frequency, type, and impact. *Journal of Palliative Medicine*.

[10] Cattagni Kleiner A., Santos-Eggimann B., Fustinoni S. (2019). Advance care planning dispositions: the relationship between knowledge and perception. *BMC Geriatrics*.

[11] Burchard R., Oikonomoulas V., Soost C., Zoremba M., Graw J. A. (2019). Indicated trauma emergency department utilization - a comparison between patients’ self-assessment and professional evaluation. *International Emergency Nursing*.

[12] Pfirstinger J., Bleyer B., Blum C., Rechenmacher M., Wiese C. H., Gruber H. (2017). Determinants of completion of advance directives: a cross-sectional comparison of 649 outpatients from private practices versus 2158 outpatients from a university clinic. *BMJ Open*.

[13] Graw J. A., Spies C. D., Kork F., Wernecke K. D., Braun J. P. (2015). End-of-life decisions in intensive care medicine-shared decision-making and intensive care unit length of stay. *World Journal of Surgery*.

[14] Van Scoy L. J., Howrylak J., Nguyen A., Chen M., Sherman M. (2014). Family structure, experiences with end-of-life decision making, and who asked about advance directives impacts advance directive completion rates. *Journal of Palliative Medicine*.

[15] Jackson J. M., Rolnick S. J., Asche S. E., Heinrich R. L. (2009). Knowledge, attitudes, and preferences regarding advance directives among patients of a managed care organization. *The American Journal of Managed Care*.

[16] Sudore R. L., Boscardin J., Feuz M. A., McMahan R. D., Katen M. T., Barnes D. E. (2017). Effect of the PREPARE website vs an easy-to-read advance directive on advance care planning documentation and engagement among veterans. *JAMA Internal Medicine*.

[17] Zafar S. N., Obirieze A., Schneider E. B. (2015). Outcomes of trauma care at centers treating a higher proportion of older patients. *Journal of Trauma and Acute Care Surgery*.

[18] Hubert E., Schulte N., Belle S. (2013). Cancer patients and advance directives: a survey of patients in a hematology and oncology outpatient clinic. *Onkologie*.

[19] Van Oorschot B., Schuler M., Simon A., Flentje M. (2012). Advance directives: prevalence and attitudes of cancer patients receiving radiotherapy. *Supportive Care in Cancer*.

[20] Pfirstinger J., Kattner D., Edinger M., Andreesen R., Vogelhuber M. (2014). The impact of a tumor diagnosis on patients’ attitudes toward advance directives. *Oncology*.

[21] Wilson D. M., Houttekier D., Kunju S. A. (2013). A population-based study on advance directive completion and completion intention among citizens of the western Canadian province of Alberta. *Journal of Palliative Care*.

[22] Marcia L., Ashman Z. W., Pillado E. B., Kim D. Y., Plurad D. S. (2018). Advance directive and do-not-resuscitate status among advanced cancer patients with acute care surgical consultation. *The American Surgeon*.

[23] Yadav K. N., Gabler N. B., Cooney E. (2017). Approximately one in three US adults completes any type of advance directive for end-of-life care. *Health Affairs*.

[24] Alano G. J., Pekmezaris R., Tai J. Y. (2010). Factors influencing older adults to complete advance directives. *Palliative and Supportive Care*.

[25] Del Pozo Puente K., Hidalgo J. L.-T., Herráez M. J. S., Bravo B. N., Rodríguez J. O., Guillén V. G. (2014). Study of the factors influencing the preparation of advance directives. *Archives of Gerontology and Geriatrics*.

[26] Rao J. K., Anderson L. A., Lin F.-C., Laux J. P. (2014). Completion of advance directives among U.S. consumers. *American Journal of Preventive Medicine*.

[27] Rurup M. L., Onwuteaka-Philipsen B. D., van der Heide A., van der Wal G., Deeg D. J. H. (2006). Frequency and determinants of advance directives concerning end-of-life care in The Netherlands. *Social Science & Medicine*.

[28] Vilpert S., Borrat-Besson C., Maurer J., Borasio G. D. (2018). Awareness, approval and completion of advance directives in older adults in Switzerland. *Swiss Medical Weekly*.

[29] Mueller L. A., Reid K. I., Mueller P. S. (2010). Readability of state-sponsored advance directive forms in the United States: a cross sectional study. *BMC Medical Ethics*.

